# Sensitivity and Specificity of SARS-CoV-2 Rapid Antigen Detection Tests Using Oral, Anterior Nasal, and Nasopharyngeal Swabs: a Diagnostic Accuracy Study

**DOI:** 10.1128/spectrum.02029-21

**Published:** 2022-02-02

**Authors:** Michael Wölfl-Duchek, Felix Bergmann, Anselm Jorda, Maria Weber, Matthias Müller, Tamara Seitz, Alexander Zoufaly, Robert Strassl, Markus Zeitlinger, Harald Herkner, Harald Schnidar, Karolina Anderle, Ulla Derhaschnig

**Affiliations:** a Department of Clinical Pharmacology, Medical University of Vienna, Vienna, Austria; b Department of Plastic, Reconstructive and Aesthetic Surgery, Medical University of Vienna, Vienna, Austria; c Department of Emergency Medicine, Medical University of Vienna, Vienna, Austria; d Fourth Medical Department for Infectious Diseases and Tropical Medicine, Clinic Favoriten, Vienna, Austria; e Faculty of Medicine, Sigmund Freud University, Vienna, Austria; f Department of Laboratory Medicine, Division of Clinical Virology, Medical University of Vienna, Vienna, Austria; g Scarletred Holding GmbH, Vienna, Austria; Johns Hopkins Hospital

**Keywords:** PCR, SARS-CoV-2, infectious disease, rapid antigen detection, sensitivity, specificity, swabbing, test performance

## Abstract

The objective of our study was to evaluate the sensitivity and specificity of rapid antigen detection tests versus those of reverse transcriptase PCR (RT-PCR) using oral, anterior nasal, and nasopharyngeal swabs. The underlying prospective, diagnostic case-control-type accuracy study included 87 hospitalized and nonhospitalized participants in a positive and a negative sample cohort between 16 March and 14 May 2021 in two hospitals in Vienna. SARS-CoV-2 infection status was confirmed by RT-PCR. Participants self-performed one oral and one anterior nasal swab for the rapid antigen test, immediately followed by two nasopharyngeal swabs for the rapid antigen test and RT-PCR by the investigator. Test results were read after 15 min, and participants completed a questionnaire in the meantime. Test parameters were calculated based on the evaluation of 87 participants. The overall sensitivity of rapid antigen detection tests versus that of RT-PCR with oral, anterior nasal, and nasopharyngeal samples was 18.18% (95% confidence interval [CI] 8.19% to 32.71%), 63.04% (95% CI 47.55% to 76.79%), and 73.33% (95% CI 58.06% to 85.4%), respectively. All sampling methods had a test specificity of 100% regardless of the cycle threshold (*C_T_*) value. Rapid antigen detection tests using self-collected anterior nasal swabs proved to be as sensitive as and more tolerable than professionally collected nasopharyngeal swabs for *C_T_* values up to 30 determined by RT-PCR. This finding illustrates the reliability of tests obtained by adequate self-collected anterior nasal specimen. Sensitivity was dependent upon the *C_T_* value for each sampling method. While the main advantage of rapid antigen detection tests is the immediate availability of results, PCR should be preferred in crucial settings wherever possible.

**IMPORTANCE** Rapid antigen detection devices for SARS-CoV-2 represent a valuable tool for monitoring the spread of infection. However, the reliability of the tests depends largely on the test performance and the respective sampling method. Nasopharyngeal swabs mark the gold standard for sample collection in suspected respiratory tract infections but are unsuitable for widespread application, as they must be performed by medically trained personnel. With the underlying study, the head-to-head test performance and the usability of self-collected samples for SARS-CoV-2 detection using rapid antigen detection devices were evaluated. The results confirm similar sensitivity of self-collected anterior nasal swabs to that of professionally collected nasopharyngeal swabs for patients with a *C_T_* of < 30 determined by RT-PCR.

## INTRODUCTION

The SARS-CoV-2 pandemic is an ongoing threat that requires effective measures, such as rapid and accurate testing methods, to contain its spread. Rapid antigen detection (RAD) tests are highly valued in the detection of potentially infectious patients, as they can provide prompt results in a cost-effective manner (1, 2). However, in terms of sensitivity, RAD tests are inferior to PCR, and test results are highly dependent on the swabbing method (3).

Nasopharyngeal (NP) swabs are typically preferred for sample collection in suspected respiratory tract infections. However, to ensure reliable results, the swab must be performed by trained medical professionals, which limits its widespread use in the general population. Moreover, NP swabs are commonly perceived as unpleasant or even painful due to the sensitivity of the nasal and nasopharyngeal mucosa. Even though complications are rare, individual preconditions or improper execution of the swab carry the risk of complications (4). As a consequence, the individual willingness to perform a NP swab might be reduced, especially in children.

Swab samples collected from the oral or anterior nasal (AN) cavity offer a less invasive alternative and may improve compliance and testing frequency. As they are easy to obtain, individuals may self-collect samples, thus eliminating the need for health care professionals and reducing the risk of virus transmission. Self-testing presents a major advance in infection control and saves costly resources in the health care sector.

Reverse transcriptase-PCR (RT-PCR) is the gold standard for laboratory diagnosis of viral respiratory infections, including SARS-CoV-2. Several studies have confirmed the reliability of saliva and samples from the AN cavity for the detection of SARS-CoV-2 RNA with PCR (5–11).

Oral swabs might offer an alternative when saliva production is impaired. However, the viral load of oral swabs obtained from SARS-CoV-2-infected children was found to be lower than that of NP swabs (12). This finding was confirmed by a recent study that compared self-collected buccal swabs and saliva samples with NP swabs (13). Buccal swabs showed poor reliability, whereas saliva samples were comparable to NP swabs.

In view of these results, the use of self-collected AN and oral samples for RAD devices is promising due to their easy collection. However, data regarding the reliability of self-collected samples for RAD devices remain limited (14–17), and the accuracy of self-collected oral swabs has yet to be investigated. Therefore, the aim of this study was to evaluate the reliability of RAD tests using self-collected oral and AN swabs and professionally collected NP swabs for detecting SARS-CoV-2 RNA using RT-PCR as reference standard.

## RESULTS

**FIG 1:**
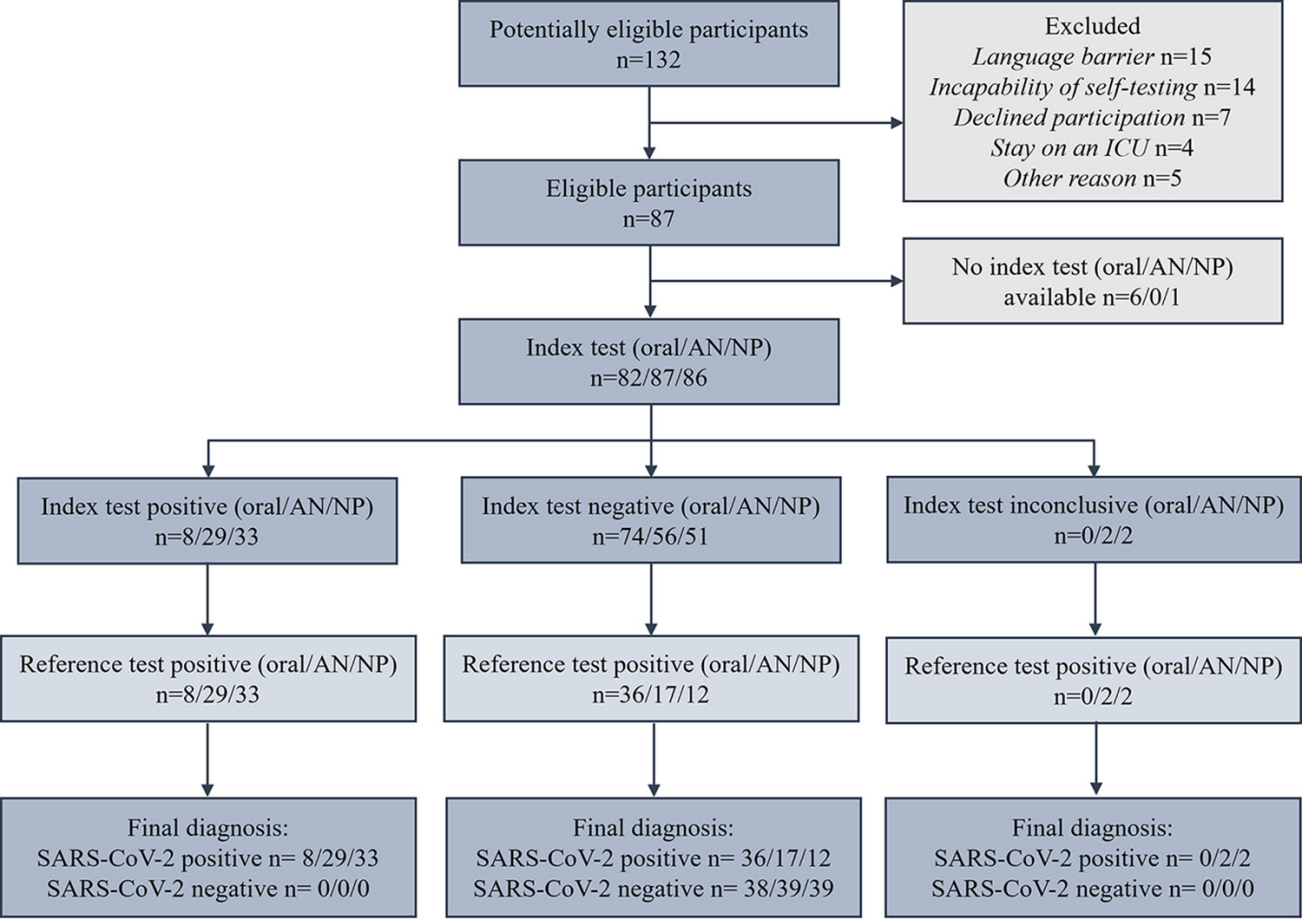
Participant flowchart. AN, anterior nasal; NP, nasopharyngeal. Index tests were treated as inconclusive if the test line of the RAD tests was barely discernible.

Demographic data are displayed in Table 1. In total, 132 participants were screened for eligibility (Fig. 1). SARS-CoV-2-positive participants were hospitalized at a regular ward and did not require intensive care.

**TABLE 1:** Demographic data of study participants^*a*^

Parameter	Hospitalized participants (*n* = 46)	Healthy participants (*n* = 41)
Age		
Age in yrs, mean (SD)	62.2 (16.1)	35.6 (13.2)
Gender		
Male (%)	27 (58.7)	22 (53.7)
Female (%)	19 (41.3)	19 (46.3)
Ethnicity		
Caucasian (%)	44 (95.6)	41 (100)
Asian (%)	1 (2.2)	0 (0)
African (%)	1 (2.2)	0 (0)
Cycle threshold		
<15 (%)	3 (6.5)	
15 to <20 (%)	6 (13.0)	
20 to <25 (%)	16 (34.8)	
25 to <30 (%)	14 (30.4)	
30 to <35 (%)	7 (15.2)	
Mean (SD)	24.2 (5.5)	
COVID-19 symptoms		
Days after symptom onset, mean (SD)	7.5 (4.6)	

a SD, standard deviation.

**FIG 2:**
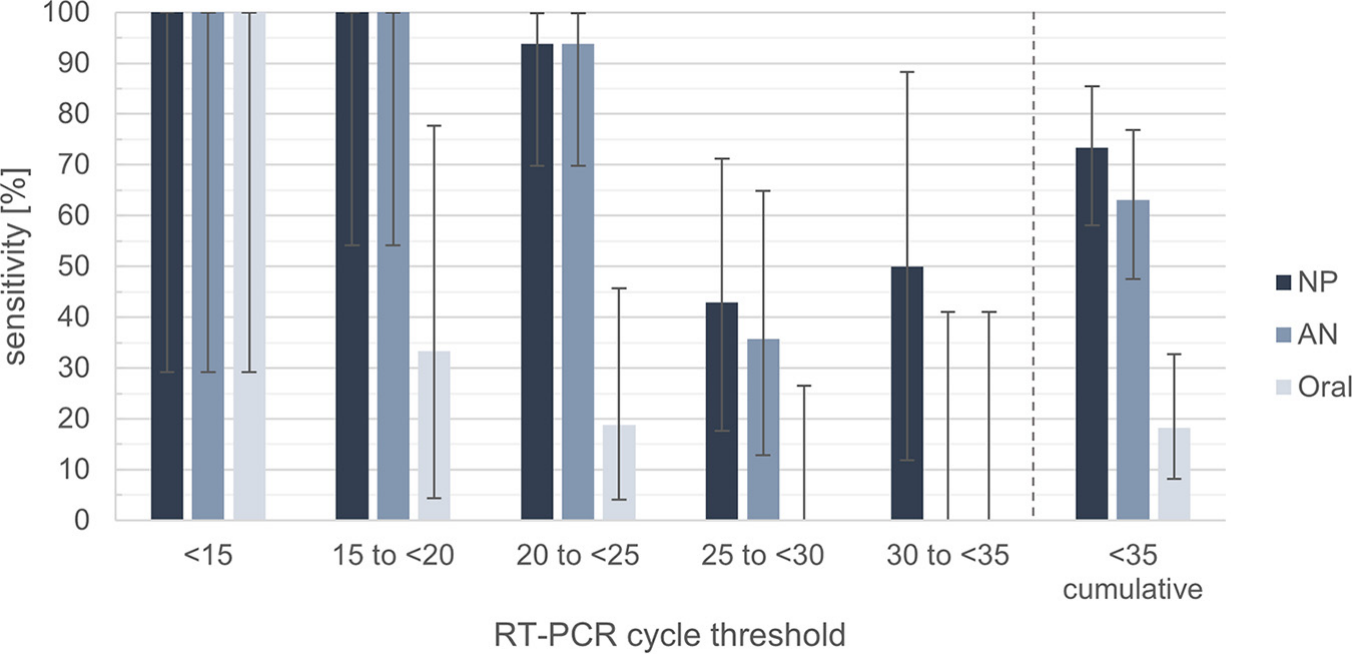
Sensitivity of RAD tests for *C_T_* value categories and cumulated *C_T_* values. AN, anterior nasal; NP, nasopharyngeal; RT-PCR, reverse transcriptase PCR.

Sensitivities of RAD tests with oral, AN, and NP samples grouped by RT-PCR-derived cycle threshold (*C_T_*) values are listed in Table 2. A decline in sensitivity was evident with higher *C_T_* values for all swabbing methods. This decline was particularly pronounced for oral samples, whose sensitivity declined from 100% for a *C_T_* of <15 to 33.33% for a *C_T_* of 15 to <20 (Fig. 2).

**TABLE 2:** Sensitivity of RAD tests for *C_T_* value categories^*a*^

Sample type	*n*	Sensitivity (%)	95% CI
*C_T_* values <15			
Oral	3	100.00	29.24–100.00
AN	3	100.00	29.24–100.00
NP	3	100.00	29.24–100.00
*C_T_* values 15 to <20			
Oral	6	33.33	4.33–77.72
AN	6	100.00	54.07–100.00
NP	6	100.00	54.07–100.00
*C_T_* values 20 to <25			
Oral	16	18.75	4.05–45.65
AN	16	93.75	69.77–99.84
NP	16	93.75	69.77–99.84
*C_T_* values 25 to <30			
Oral	12	0.00	0.00–26.46
AN	14	35.71	12.76–64.86
NP	14	42.86	17.66–71.14
*C_T_* values 30 to <35			
Oral	7	0.00	0.00–40.96
AN	7	0.00	0.00–40.96
NP	6	50.00	11.81–88.19
All *C_T_* values			
Oral	44	18.18	8.19–32.71
AN	46	63.04	47.55–76.79
NP	45	73.33	58.06–85.40

a AN, anterior nasal; NP, nasopharyngeal; CI, confidence interval; *C_T_*, cycle threshold.

All sampling methods had an equal specificity of 100% regardless of the *C_T_* value (Table 3). Positive predictive value (PPV) was 100%, as there were no false-positive tests reported.

**TABLE 3:** Diagnostic test parameters of RAD devices^*a*^

Sample type	*n*	Sensitivity	Specificity	PPV	NPV	Accuracy
Oral	82	18.18	100.00	100.00	51.35	56.10
95% CI		8.19–32.71	90.75–100.00	NA	47.87–54.82	44.70–67.04
AN	87	63.04	100.00	100.00	70.69	80.46
95% CI		47.55–76.79	91.40–100.00	NA	62.31–77.86	70.57–88.19
NP	86	73.33	100.00	100.00	77.36	86.05
95% CI		58.06–85.40	91.40–100.00	NA	67.79–84.72	76.89–92.58

a AN, anterior nasal; NP, nasopharyngeal; CI, confidence interval; PPV, positive predictive value; NPV, negative predictive value; NA, not applicable.

**FIG 3:**
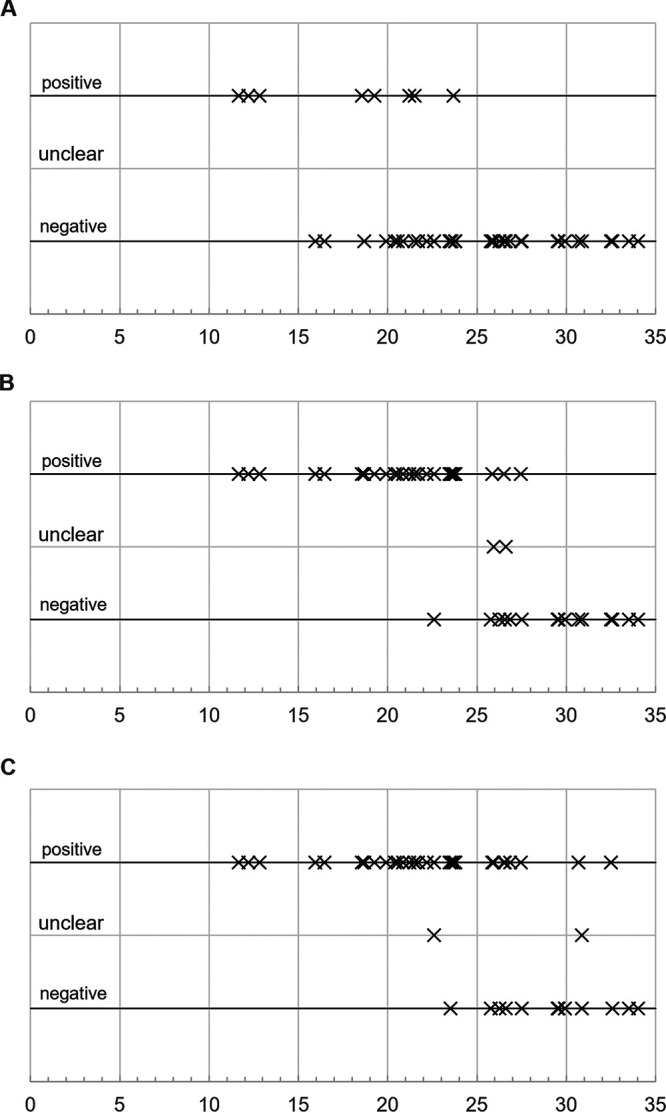
Individual test results. Test results and respective RT-PCR *C_T_* value for (A) self-collected oral swab, *n* = 44 (8/36), (B) self-collected anterior nasal swab, *n* = 46 (33/13), (C) investigator-collected nasopharyngeal swab, *n* = 45 (33/12). Rapid antigen detection devices with faint test lines produced unclear results that were ultimately deemed positive.

Parameters of the test comparison with regard to professionally collected NP swabs are listed in Table 4. Individual test results of self-collected oral, self-collected AN, and investigator-collected NP swabs were positive in 18% (8 of 44), 72% (33 of 46), and 73% (33 of 45) of cases (Fig. 3).

**TABLE 4:** Comparison of NP swabs and self-collected oral and AN swabs for RAD devices^*a*^

Sample type	*n*	PPA	NPA	ORA
Oral	44	24.24	100.00	43.18
95% CI		11.09–42.26	71.51–100.00	28.35–58.97
AN	45	81.82	83.33	82.22
95% CI		64.54–93.02	51.59–97.91	67.95–92.00

a AN, anterior nasal; CI, confidence interval; PPA, positive percentage agreement; NPA, negative percentage agreement; ORA, overall rates of agreement.

Although most RAD devices delivered conclusive outcomes with bright red control and test lines on the test cassette, four RAD devices produced barely visible, faint test lines that were ultimately deemed positive. Missing test results were excluded from the analysis.

Questionnaire information assessing discomfort and pain ratings indicated a significant difference in the perception of swabbing methods in all participants. Least discomfort and pain were associated with self-collected oral swabs as illustrated in Fig. S1 and S2. Subjective ratings of discomfort and pain appeared to affect the participants’ willingness to frequently perform the respective test methods, as displayed in Fig. S3.

Swab quality of patients’ self-collected AN swabs assessed by the investigator was classified as “good” in 39 of 46 (84.78%) cases, compared to 32 of 46 (69.57%) cases in oral swabs (Fig. S4). Support was provided in 15 of 46 (32.61%) and 25 of 46 (54.35%) cases for AN and oral swabs, respectively (Fig. S5).

No adverse event was reported for any sampling method.

## DISCUSSION

In participants with a *C_T_* of <30, RAD tests using self-collected AN swabs were similarly sensitive to RAD devices using professionally collected NP swabs.

Based on the findings of several previous studies, viable virus cultivation is achieved more often when *C_T_* values are below 25 but becomes increasingly unlikely when they exceed 30, which goes in hand with infectivity (18–23).

With reference to professionally collected NP swabs, RAD devices using AN swabs performed comparably. These results are in good agreement with the test performance of AN swabs conducted by medically trained personnel observed in previous studies (14, 15). In line with these findings, sensitivity of RAD devices was shown to be comparable to RT-PCR using professionally collected AN swabs, emphasizing the value of AN swabs for screening purposes when used for RAD devices (16, 17).

In contrast, self-collected oral swabs had a relatively low overall sensitivity of 18% but a specificity of 100%. With reference to professionally collected NP swabs, overall positive percentage agreement (PPA) and negative percentage agreement (NPA) of RAD devices using oral swabs were 24% and 100%, which is well comparable to the test performance of saliva samples reported by Agulló et al., thus rendering oral swabs unsuitable for SARS-CoV-2 RAD devices (14).

Based on the above evidence, RAD tests require considerable viral loads to deliver reliable results. While positive test results thus detect an infection with a high degree of certainty, negative results must not be interpreted as ruling out an infection.

In addition, the course of viral load and that of antigen response are variable and dynamic throughout the course of infection (24). As a consequence, the antigen load may be below the limit of detection of antigen-based assays, especially during early infection.

Currently, RT-PCR is the most sensitive diagnostic method, allowing the detection of small amounts of the viral genome, and thus has potential to confirm infection at any stage of the disease. Because of this property, RT-PCR may be the preferred method in critical settings, such as health facilities and nursing homes. RAD devices, on the other hand, should be considered complementary, high-threshold tools that play their strengths especially in patients with a high pretest probability, i.e., symptomatic patients or close contacts of infected patients.

Usability of self-collected samples for SARS-CoV-2 detection using RAD devices has been addressed by few studies (6, 25). Similarly, only few studies have investigated the tolerability and quality of different sample methods (26, 27). In the underlying study, participants were observed and completed a questionnaire on tolerability, painfulness, and willingness to frequently perform a swabbing method immediately after swabbing, therefore providing relevant information on their perception.

A difference in sampling quality of hospitalized and healthy participants was observed, which is also reflected by the number of hospitalized participants that required support by the supervising investigator.

Considering that hospitalized and healthy participants received identical instructions, one possible explanation for this discrepancy could be the abnormal situation of hospitalization. In addition, the age difference between the study groups might also have contributed to these differences. Nevertheless, clear instructions are essential to obtain high-quality samples, which in turn form the basis for reliable test results.

According to the questionnaire, participants showed a greater willingness to perform AN swabs regularly than to perform NP swabs. For this reason, AN swabs might be a more beneficial screening method despite the slightly lower sensitivity of RAD tests using AN swabs compared to that of RAD tests using NP swabs.

A limitation of this study is the moderate sample size for the evaluation of test specificity.

Also, participants were supported if they were unable to perform the swab correctly, opposing a real-life-scenario. However, this intervention was necessary to assess the test performance, which was the primary aim of this study.

Additionally, the mean age difference between the study groups may have influenced the performance and quality of the swabs. Older age and multimorbidity are undoubtedly among the greatest risk factors for severe SARS-CoV-2 infection requiring hospitalization, which resulted in a higher average age in the patient population but did not affect the primary endpoint (28–30).

We also acknowledge the difficulties in objectively assessing factors such as perception of swabs and willingness to frequently repeat sampling.

**Conclusion.** Self-collected AN swabs demonstrated sensitivity similar to that of professionally collected NP swabs in participants with *C_T_* of <30, illustrating the reliability of RAD tests obtained by using adequate self-collected AN specimens.

Self-collected AN and oral swabs were found to be more tolerable and less painful than NP swabs. Although RAD tests using self-collected oral swabs were perceived to be most tolerable and least painful, they were sensitive only in participants with *C_T_* of <15.

However, RAD test results are highly dependent on the particular swabbing method and are inferior to RT-PCR in terms of sensitivity, limiting its advantages of rapid and cost-effective results to patients with increased pretest probability.

## MATERIALS AND METHODS

### Study design.

This prospective, diagnostic case-control-type accuracy study was conducted in accordance with the ICH-GCP guidelines, the current version of the Declaration of Helsinki (October 2013), and the Austrian Medical Devices Act (Medizinproduktegesetz; MPG). Ethical approval for this study was obtained from the Ethics Committee of the Medical University of Vienna. The study was conducted at the General Hospital, Vienna and Clinic Favoriten, Vienna in Austria.

### Study population and target condition.

The study included 46 hospitalized participants in a positive sample group with active SARS-CoV-2 infection and 41 healthy participants in a negative sample group, both confirmed by RT-PCR in a consecutive series. Participants were enrolled at the Medical University of Vienna and the Clinic Favoriten, Vienna, between 16 March and 14 May 2021, after giving oral and written informed consent to participate.

### Sample collection.

Oral and AN swabs were self-collected by study participants after they received written instructions. Additional explanations were provided only if a participant had difficulties performing the sampling procedure correctly. All swabs were performed at least 30 min after eating, drinking, or brushing teeth.

Oral samples were obtained by swabbing between the gums and the inside of the cheeks, collecting saliva and mucosa from either side of the oral cavity. Nasal swabs were self-collected from both nostrils by gently twisting and pushing the swab against the inner wall of the nasal vestibule. NP swabs for RAD devices and RT-PCR tests were performed by the investigating physician immediately after by slightly tilting the participant’s head back and inserting the swab through the nostril parallel to the palate. The swab was then gently rotated at the wall of the nasopharyngeal cavity to absorb secretions before being removed in a rotating motion. At the time the index tests were performed, reference test results were not available to the investigators.

### SARS-CoV-2 antigen test (index test).

For RAD devices, the Medomics SARS-CoV-2 antigen test device (Jiangsu Medomics Medical Technology Co., Ltd., Nanjing, Jiangsu, China) with a limit of detection (LoD) of 10 50% tissue culture infective doses (TCID_50_)/mL for SARS-CoV-2 was used. This lateral flow assay is based on colloidal gold immunochromatography using the double-antibody-sandwich method.

Collected swabs were inserted into and pressed against the inner wall of the lysis buffer tubes to dissolve the samples. Approximately 100 µL (4 drops) was then dropped into the sample well of the antigen testing device. A visible red test and control line indicated a positive result. RAD test results were read after 15 min and were then photodocumented using Scarletred Vision medical device software (Scarletred Holding GmbH, Vienna, Austria).

### RT-PCR (reference standard).

For RT-PCR testing, NP swabs were performed by the investigators immediately after self-collection of oral and AN swabs. The collected material was diluted in 2 mL of physiologic saline solution and was immediately sent to the study site’s virological laboratory for RT-PCR testing. Samples were analyzed by the laboratory without knowledge of the participant’s infectious status. RT-PCR was performed on the fully automated CE/IVD-certified Roche Cobas 6800 RT-PCR system (Roche Diagnostics GmbH, Forrenstrasse 2, 6343 Rotkreuz ZG, Switzerland) with the CE/IVD-certified Cobas SARS-CoV-2 assay, according to the manufacturer’s instructions. The Cobas SARS-CoV-2 assay targets highly conserved open reading frame 1a/b (ORF-1a/b) and E-gene regions and uses 400 µL of sample processing volume. Viral load was determined in a semiquantitative manner expressed by the number of PCR cycles needed to amplify RNA to a detectable level, termed cycle threshold (*C_T_*), with a test positivity cutoff of *C_T_* less than 35.

### Statistical analysis.

Statistical analysis was performed using version 25 of the commercially available computer program IBM SPSS Statistics (IBM Corp., Armonk, NY, USA) and Microsoft Excel Version 2016 (Microsoft Corporation, Redmond, WA, USA).

The sample size consideration is based on exact binomial 95% confidence intervals (95% CI) for the estimated diagnostic test sensitivity of 87%. To yield a lower bound of the confidence interval above 74%, 46 reference test positive individuals needed to be included.

Sensitivity and specificity of the index tests, i.e., RAD tests using oral, AN, and NP swabs, are based on comparison to RT-PCR results acting as the reference standard. In this study, sensitivity describes the RAD test’s ability to identify participants whose RT-PCR result confirms SARS-CoV-2 infection. Analogously, specificity refers to the RAD test’s ability to identify participants with negative RT-PCR test results. Positive (PPV) and negative predictive value (NPV) indicate the probability of SARS-CoV-2 infection in participants with a positive test result and no SARS-CoV-2 infection in participants with a negative test result, respectively.

For comparison to a test that is not considered the reference standard, positive (PPA) and negative percentage agreements (NPA) are appropriate instead of sensitivity and specificity; however, calculations are identical. Accordingly, percentage agreements were used to describe the test performance of RAD tests using self-collected oral and AN swabs compared to that of RAD tests using professionally collected NP swabs.

Accuracy describes the test’s ability to discriminate healthy and infected participants correctly with regard to the reference standard. If the index test is compared to a test that is not the reference standard, the overall rate of agreement (ORA) is used as an equivalent for accuracy.

Test performance parameters were calculated according to the following equations:

$$\text{sensitivity}/\text{PPA}=\frac{\mathrm{TP}}{\mathrm{TP + FN}}$$

$$\text{specificity}/\text{NPA }=\frac{\mathrm{TN}}{\mathrm{TN + FP}}$$

$$\text{PPV}=\frac{\mathrm{TP}}{\mathrm{TP + FP}}$$

$$\text{NPV}=\frac{\mathrm{TN}}{\mathrm{TN + FN}}$$

$$\text{accuracy}=\frac{\mathrm{TP + TN}}{\mathrm{TP + TN + FP + FN}}$$

where true positive (TP) is index test positive, reference test/comparator positive, true negative (TN) is index test negative, reference test/comparator negative, false positive (FP) is index test positive, reference test/comparator negative, and false negative (FN) is index test negative, reference test/comparator positive. Ninety-five percent confidence intervals for test performance parameters were calculated using exact Clopper-Pearson method.

Wilcoxon signed rank test was applied for ordinally scaled paired variables retrieved from the questionnaires, in particular information on discomfort, pain, and willingness to frequently perform a sampling method. The two-sided significance level α was set at 0.05.
