# Supplementary material for: Sensitivity and Specificity of SARS-CoV-2 Rapid Antigen Detection Tests Using Oral, Anterior Nasal, and Nasopharyngeal Swabs: a Diagnostic Accuracy Study
Source: Microbiol Spectr. 2022 Feb 2;10(1):e02029-21. doi: 10.1128/spectrum.02029-21 (PMC8809344; doi:10.1128/spectrum.02029-21)
Supplement: SUPPLEMENTAL FILE 1 — Supplemental material. Download SPECTRUM02029-21_Supp_1_seq10.pdf, PDF file, 0.3 MB [file spectrum02029-21_supp_1_seq10.pdf]

Supplementary Appendix

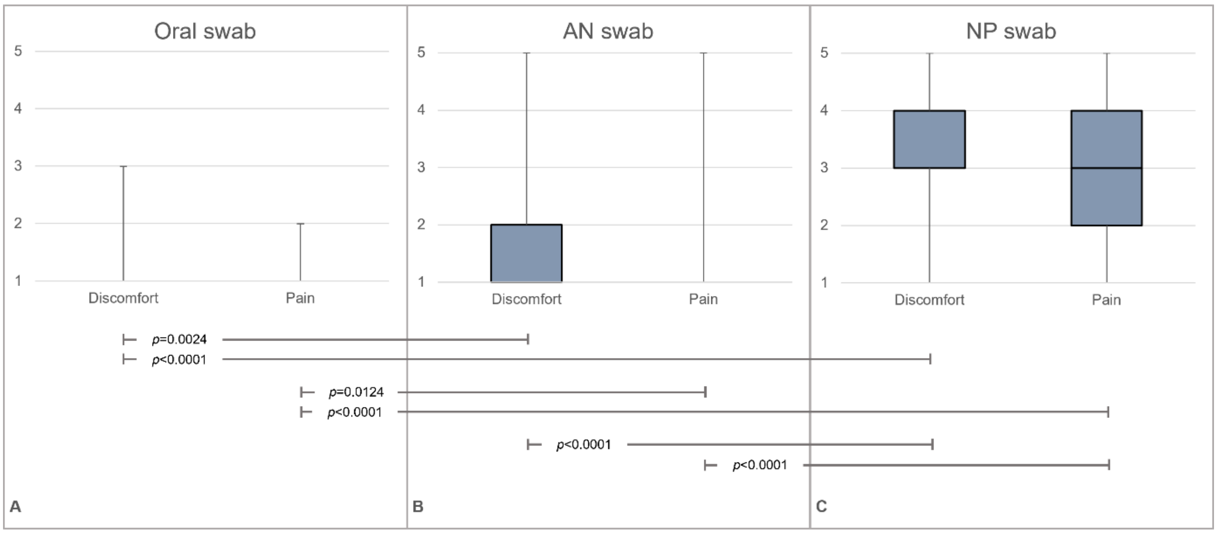

Figure S1: Discomfort and pain ratings in hospitalized participants.

AN, anterior nasal; NP, nasopharyngeal.

A questionnaire assessed severity from 1 to 5, whereas 1=least discomfort or pain and 5=most discomfort or pain.Results are displayed as minimum/1st quartile/median/3rd quartile/maximum.

(A) self-collected oral swab – discomfort and pain (1/1/1/1/3) and (1/1/1/1/2),

(B) selfcollected anterior nasal swab – discomfort and pain (1/1/1/2/5) and (1/1/1/1/5),

(C) professionally collected nasopharyngeal swab – discomfort and pain (1/3//4/4/5) and (1/2/3/4/5).

p-values were calculated using the Wilcoxon signed rank test.

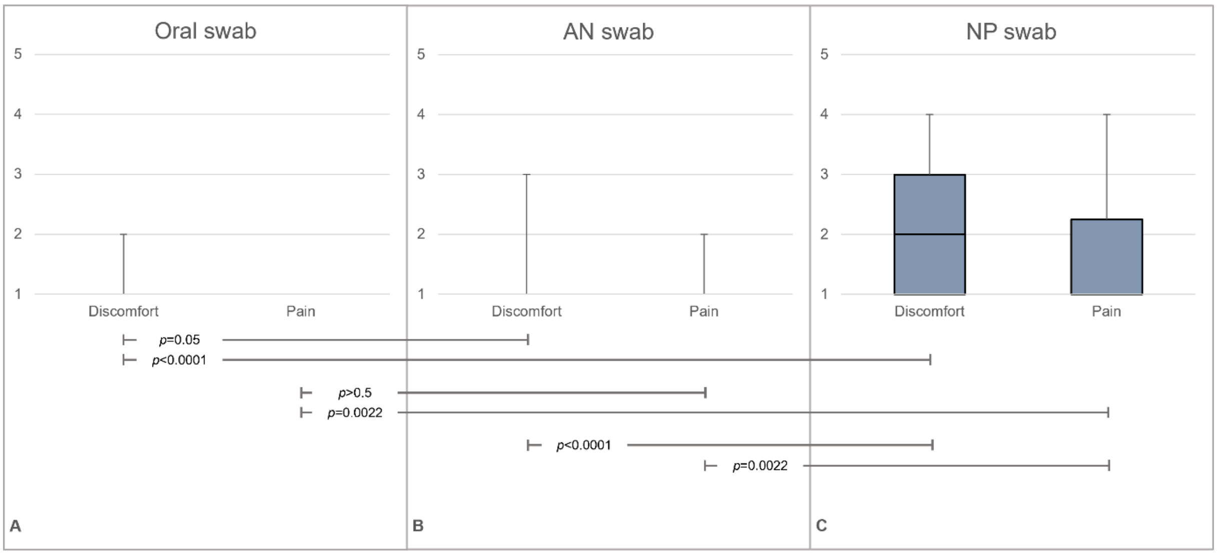

Figure S2: Discomfort and pain ratings in healthy volunteers.

AN, anterior nasal; NP, nasopharyngeal.

A questionnaire assessed severity from 1 to 5, whereas 1=least discomfort or pain and 5=most discomfort or pain. Results are displayed as minimum/1st quartile/median/3rd quartile/maximum.

(A) self-collected oral swab – discomfort and pain (1/1/1/1/2) and (1/1/1/1/1),

(B) self-collected anterior nasal swab – discomfort and pain (1/1/1/1/3) and (1/1/1/1/2),

(C) professionally collected nasopharyngeal swab – discomfort and pain (1/1//2/3/4) and (1/1/1/2.25/4).

p-values were calculated using the Wilcoxon signed rank test.

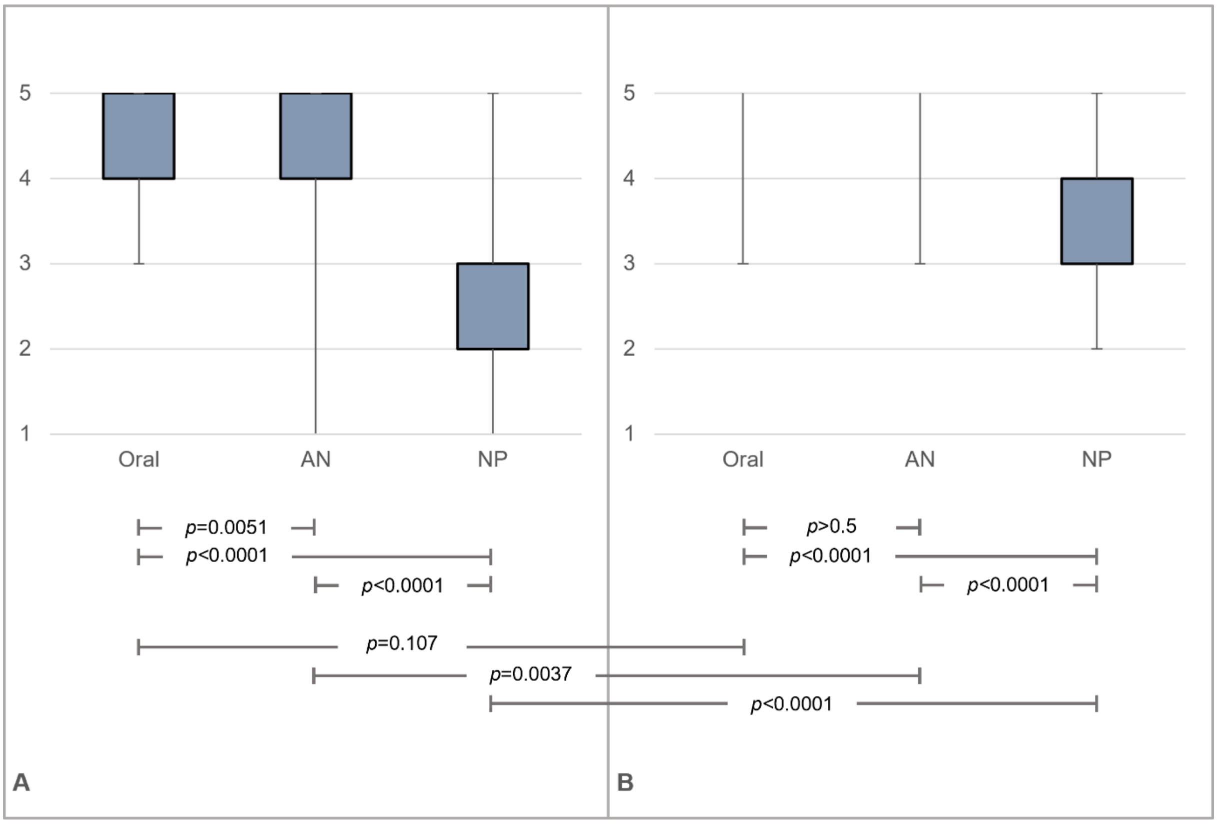

Figure S3: Willingness to frequently perform different swabbing methods.

AN, anterior nasal; NP, nasopharyngeal; A questionnaire assessed willingness to frequently perform self-collected oral and anterior nasal and professionally collected nasopharyngeal swabs in hospitalized participants (A) and healthy volunteers (B), whereas 1=unwillingly to frequently perform the swab and 5=strong willingness to frequently perform the swab. Results are displayed as minimum/1st quartile/median/3rd quartile/maximum.

(A) oral (3/4/5/5/5), anterior nasal (1/4/5/5/5), nasopharyngeal (1/2/2/3/5).

(B) oral (3/5/5/5/5), anterior nasal (3/5/5/5/5), nasopharyngeal (2/3/4/5/5).

p-values were calculated using the Wilcoxon signed rank test.

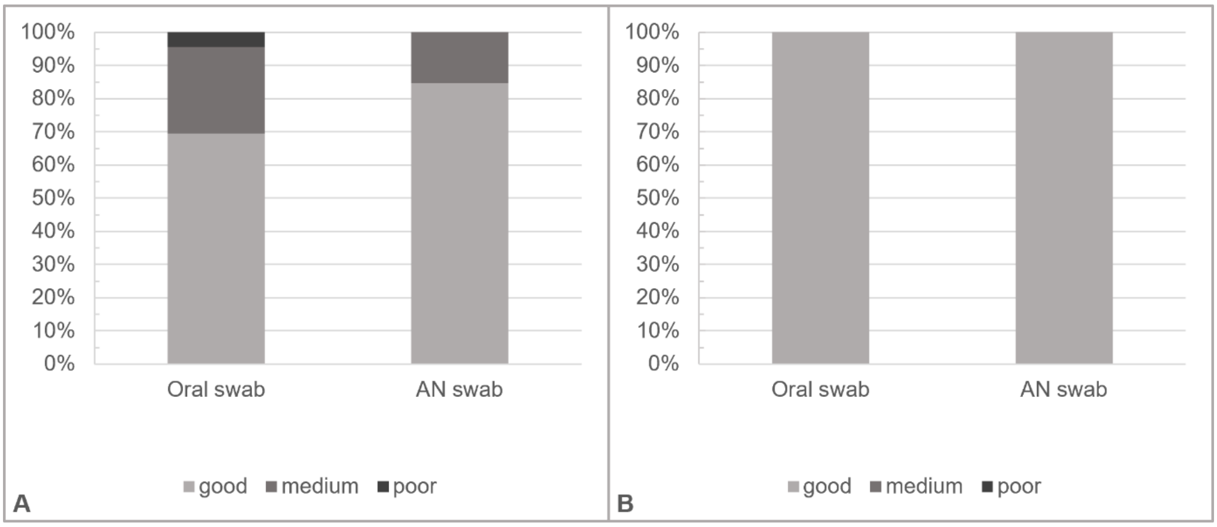

Figure S4: Swab quality.

AN, anterior nasal.

Swab quality was assessed by the supervising investigator during sample collection and classified as good, medium or poor.

(A) self-collected oral and anterior nasal swabs in hospitalized participants (69.57%/26.09%/4.35%) and (84.78%/15.22%/0%),

(B) self-collected oral and anterior nasal swabs in healthy volunteers (100%/0%/0%) and (100%/0%/0%).

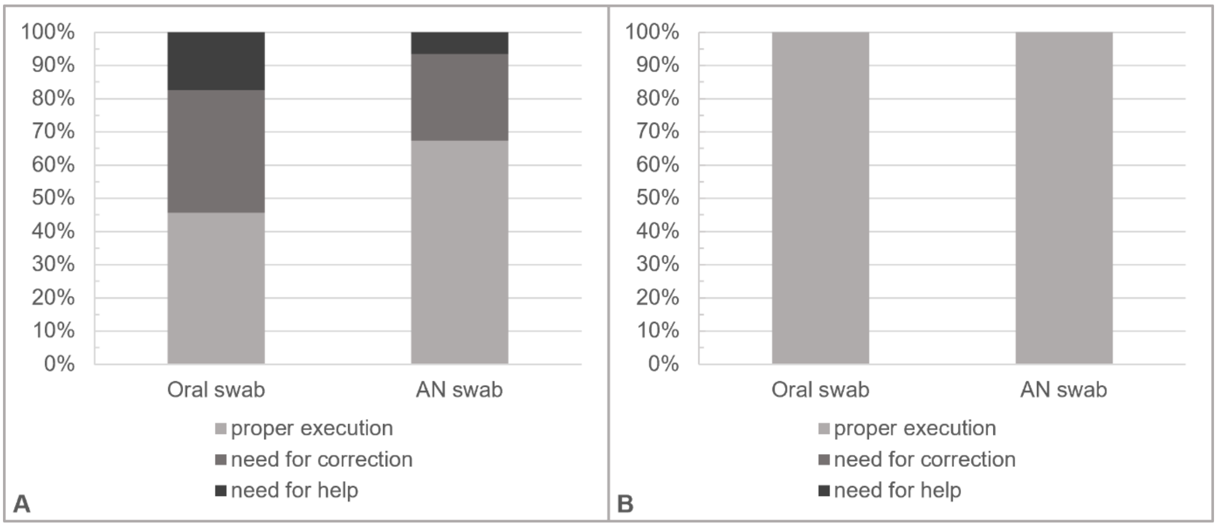

Figure S5: Execution of swabbing.

AN, anterior nasal.

Oral and anterior nasal swab execution and requirement of support (proper execution/need for correction/need for help) were documented by the supervising investigator during sample collection.

(A) self-collected oral and anterior nasal swabs in hospitalized participants (45.65%/36.96%/17.39%) and (67.39%/26.09%/6.52%),

(B) self-collected oral and anterior nasal swabs in healthy volunteers (100%/0%/0%) and (100%/0%/0%)
